# The Effects of Tobacco Coverage in the Public Communication Environment on Young People’s Decisions to Smoke Combustible Cigarettes^†^

**DOI:** 10.1093/joc/jqab052

**Published:** 2022-01-13

**Authors:** Robert Hornik, Steven Binns, Sherry Emery, Veronica Maidel Epstein, Michelle Jeong, Kwanho Kim, Yoonsang Kim, Elissa C Kranzler, Emma Jesch, Stella Juhyun Lee, Allyson V Levin, Jiaying Liu, Matthew B O’Donnell, Leeann Siegel, Hy Tran, Sharon Williams, Qinghua Yang, Laura A Gibson

**Affiliations:** Annenberg School for Communication, University of Pennsylvania, Philadelphia, PA 19104, USA; Social Data Collaboratory, NORC-University of Chicago, Chicago, IL 60637, USA; Social Data Collaboratory, NORC-University of Chicago, Chicago, IL 60637, USA; Social Data Collaboratory, NORC-University of Chicago, Chicago, IL 60637, USA; Annenberg School for Communication, University of Pennsylvania, Philadelphia, PA 19104, USA; Department of Health Behavior, Society and Policy, Rutgers University School of Public Health, Piscataway, NJ 08854, USA; Annenberg School for Communication, University of Pennsylvania, Philadelphia, PA 19104, USA; Department of Communication, Cornell University, Ithaca, NY 14850, USA; Social Data Collaboratory, NORC-University of Chicago, Chicago, IL 60637, USA; Annenberg School for Communication, University of Pennsylvania, Philadelphia, PA 19104, USA; Fors Marsh Group, Arlington, VA 22201, USA; Annenberg School for Communication, University of Pennsylvania, Philadelphia, PA 19104, USA; Annenberg School for Communication, University of Pennsylvania, Philadelphia, PA 19104, USA; Department of Media and Communication, Konkuk University, Seoul, South Korea; Annenberg School for Communication, University of Pennsylvania, Philadelphia, PA 19104, USA; Department of Communication, Villanova University, Villanova, PA 19085. USA; Annenberg School for Communication, University of Pennsylvania, Philadelphia, PA 19104, USA; Department of Communication Studies, University of Georgia, Athens, GA 30602, USA; Annenberg School for Communication, University of Pennsylvania, Philadelphia, PA 19104, USA; Annenberg School for Communication, University of Pennsylvania, Philadelphia, PA 19104, USA; Tobacco Control Research Branch, National Cancer Institute, Bethesda, MD 20814, USA; Social Data Collaboratory, NORC-University of Chicago, Chicago, IL 60637, USA; Annenberg School for Communication, University of Pennsylvania, Philadelphia, PA 19104, USA; School of Information, University of California, Berkeley. Berkeley, CA 94704, USA; Annenberg School for Communication, University of Pennsylvania, Philadelphia, PA 19104, USA; Department of Communication Studies, Texas Christian University, Fort Worth, TX 76129, USA; Annenberg School for Communication, University of Pennsylvania, Philadelphia, PA 19104, USA; Department of Medical Ethics and Health Policy, Perelman School of Medicine, University of Pennsylvania, Pennsylvania, PA 19104, USA

**Keywords:** Automated Coding, Media Effects, Cigarettes, Twitter, YouTube, Public Communication Environment

## Abstract

In today’s complex media environment, does media coverage influence youth and young adults’ (YYA) tobacco use and intentions? We conceptualize the “public communication environment” and effect mediators, then ask whether over time variation in exogenously measured tobacco media coverage from mass and social media sources predicts daily YYA cigarette smoking intentions measured in a rolling nationally representative phone survey (N = 11,847 on 1,147 days between May 2014 and June 2017). Past week anti-tobacco and pro-tobacco content from Twitter, newspapers, broadcast news, Associated Press, and web blogs made coherent scales (thetas = 0.77 and 0.79). Opportunities for exposure to anti-tobacco content in the past week predicted lower intentions to smoke (Odds ratio [OR] = 0.95, p < .05, 95% confidence interval [CI] = 0.91–1.00). The effect was stronger among current smokers than among nonsmokers (interaction OR = 0.88, p < .05, 95% CI = 0.77–1.00). These findings support specific effects of anti-tobacco media coverage and illustrate a productive general approach to conceptualizing and assessing effects in the complex media environment.

Does the public communication environment (PCE), reflecting messages from multiple sources including both newer (e.g., Twitter, web blogs) and more traditional media coverage (e.g., newspapers, broadcast news), affect young people’s decisions to smoke combustible cigarettes? The answer to this question has deep public health significance given the toll of smoking. It also has significance for communication research. There is a long tradition of studying the effects of media exposure on beliefs and behavior (cf. Potter & Riddle, 2007). A particular concern in that tradition is undertaking studies that accurately represent the real world while still making strong causal claims. We begin this article with some discussion of the complexities of conceptualizing and estimating media effects, and then turn to the presentation of our study, an explicit response to these complexities.

Some media effects studies isolate the influence of single messages, or of a class of messages, from one source (e.g., entertainment television, Kim & Vishak, 2008; Moyer-Gusé & Nabi, 2010; Ward & Rivadeneyra, 1999) or on one theme (e.g., television violence, Bushman & Huesmann, 2006; Paik & Comstock, 1994; Wood, Wong, & Chachere, 1991). These focused studies often benefit from experimental frameworks in which causal claims can be strong, and media exposures can be reasonably isolated from their context. In contrast, if one wants to understand the messy effects that occur when (a) exposure to clusters of related themes happens over time, (b) information comes from a range of potentially reinforcing sources, and (c) messages are processed though social networks and institutional contexts, then experimental approaches which hold the messy context constant are less informative.

We address that challenge in this article. We argue that media effects naturally occur in the context of such a complex PCE, where people are immersed in messages from multiple sources, and make behavioral choices reflecting their engagement with that environment. Estimates of these sorts of media effects need to respect the fact that people live in such a PCE. This article estimates the effects of a naturally occurring, multi-faceted PCE regarding a specific behavior—smoking—and it elaborates a methodological approach for making such claims.

## The idea of the PCE and its conceptualized effects

Before describing research that uses related approaches, we elaborate the idea of the PCE and our hypothesis of how it might affect behavior. Immersion in a PCE (vs. exposure to a single message) may affect a person’s beliefs and behaviors through a range of pathways (cf. Hornik, 2002; Hornik & Yanovitzky, 2003). Repeated message coverage across multiple sources may (a) provide multiple exposures, increasing the likelihood for learning content, (b) make it more likely that content is available when an individual is ready to engage with it, (c) increase the salience of (or prime) an argument so individuals are more likely to weigh it when they make a decision, (d) imply the message has normative legitimacy because multiple sources are offering consistent messages, and (e) increase attention to a topic, which in turn may make it more likely to be addressed in personal conversations. These multiple routes of effect highlight two aspects of the PCE likely to affect belief and behavioral outcomes: the sheer volume of messages relevant to a behavior because multiple sources address it (quantity), and the degree of coherence across sources in how they address the topic (consistency).

The specific question for this article is whether the PCE around tobacco (and combustible cigarettes, particularly) influences youth and young adult decisions to initiate smoking, or, if they are current smokers, to quit smoking. The conceptual and methodological approach taken here may also be relevant to broader efforts to assess media effects in the current complex media environment.

A common nonexperimental approach relies on observational (often survey) data, where individuals report their exposure to media sources and their beliefs and behaviors, the hypothesized effects of their exposure (e.g., Charlesworth & Glantz, 2005; Durkin, Brennan, & Wakefield, 2012; Forsyth, Kennedy, & Malone, 2013; Lovato, Watts, Stead, & Cochrane Tobacco Addiction Group, 2011; Wakefield, Loken, & Hornik, 2010). This type of approach makes causal claims about media effects by assessing the cross-sectional association of exposure measures and outcomes, often statistically adjusting for potential confounders. However, methodologists are often concerned about such approaches because of endogeneity—referring to the use of a common measurement method for the predictor and outcome. Endogeneity of study measures increases uncertainty about the causal order between putative cause and effect, and opens the possibility that an observed association may actually reflect the influence of unmeasured confounders (Prior, 2009a, 2009b).

When wanting to make causal claims, researchers have made important efforts to resolve concerns about endogeneity; examples include using naturalistic field experiments, instrumental variable designs, and longitudinal studies including time series and fixed effects (Angrist & Pischke, 2008; Morgan & Winship, 2015). Each of those approaches has important strengths and makes sense in particular contexts. Here we use a different approach to reduce the threats to causal inference associated with endogeneity. We rely on exogenous measurement for media exposure, which is not contingent on the reports of individual respondents. If measurements of media exposure are independent of measurements of the outcome (i.e., if they have no common method covariance), then it is more likely that an observed association reflects the influence of one variable on the other, rather than the influence of some individual-level confounder on both variables. If we can make the additional plausible argument that the media variable could not have been caused by the outcome variable, then our uncertainty about causal claims is greatly reduced (Liu & Hornik, 2016; Slater, 2004).

Our broad approach was to measure all coverage about tobacco (both intentionally persuasive and simply descriptive) across multiple new and old media sources, each day over 3 years, and to describe how that content varies over time in terms of the quantity of anti- and pro-tobacco content. In parallel, we surveyed a rolling cross-sectional representative national sample of 13- to 25-year-olds every day over the same period (new samples released weekly). We then asked whether variation in the previous 7 days of content is associated with variation in each day’s survey-measured intentions to smoke in the next 6 months.

The volume and coherence of tobacco messages in the PCE can vary across time, across geographic units, or across individuals with different media diets. In the current study, we used time as our analytic unit, with 1,140 days of interviews matched to prior periods of coverage. We assigned each respondent PCE scores based on the relative frequency of tobacco media coverage across multiple sources in the past week or in the past 4 weeks.

Two substantial assumptions are embedded in the decision to represent PCE scores as aggregated from the measured content of multiple sources. First, we assume the aggregated measures are the best indicators of what is being discussed broadly in the PCE, i.e., not just in the measured sources (e.g., Twitter, U.S. newspapers) but also in those which we did not measure (e.g., Instagram, personal conversations). Second, we assume that as the PCE varies in its engagement with tobacco, average individual exposure to such information varies coordinately. Therefore, the content measures, which reflect opportunities for exposure, are assumed to be suitable proxies for direct measures of individual exposure.

Messages in the PCE are not necessarily homogeneous in their general support or opposition of a behavior. For example, although for some of the sources, the dominant position on combustible cigarette use in the current study is that it is an unhealthy behavior, this is not necessarily true for all sources (Kim et al., 2020). There can be more balance in the stance toward a topic between sources, or even within a source. Some content encourages a behavior, and some discourages it. As Fan (2002) has noted, much media content presents both pro and anti-views, and the effect of pro- and anti-content coverage on individual health behaviors may be different. Our study separately estimates the amounts of pro- and anti-coverage across time.

## Conceptual arguments underpinning this study

This study specifically tests whether pro- or anti-tobacco messages found in routine media content are associated with tobacco beliefs and intentions. This question may appear to be theoretically unengaged. However, the design, methods, and analyses reflect essential and specific conceptual choices. We elaborate those ideas here, first outlining likely mediational processes for the effects we hypothesize and then elaborating the fundamental theoretical expectations underpinning those choices. In some cases, we provide evidence related to those mediational processes and theoretical expectations.

The essential hypothesis of this study is that variation in opportunities for exposure to valenced messages produces changes in intentions to smoke (which, in turn, affects smoking behavior). It is legitimate to ask then what mediational process might produce such an effect and whether we have the ability to test a particular mediational process. Following Hornik and Yanovitzky (2003), we categorize mediational paths into three broad types: institutional effects, social network effects, and direct cognitive effects. Then, relying on the arguments of Fishbein and Ajzen (2010), Cialdini, Reno, and Kallgren (1990), and Bandura (2001) among other behavioral theorists, we break down direct cognitive effects to include beliefs about expected outcomes, descriptive norm beliefs, and self-efficacy beliefs.

Institutional effects include media coverage influences on the wide range of agencies that individuals interact with, which, in turn, influence individual behavior (cf. Yanovitzky, 2002). For example, media coverage of anti-smoking content might lead state legislators to consider tighter regulation of tobacco access or increases in tobacco excise taxes; tightened access rules and higher taxes might, in turn, reduce individual interest in smoking. These effects, importantly, do not require direct exposure to coverage by individual smokers; variation in the PCE can influence legislators directly and produce individual effects, indirectly. If this path operated, one might expect an association between PCE variation over time and individual behavior, although the effect would likely be cumulative and lagged and vary substantially across locations with distinct policy environments. The approach we take in this study, focusing on overall short-term associations with 7- or 28-day lags, might not be sensitive to such effects.

Social network effects, like institutional effects, do not assume that the PCE affects individual behavior through direct exposure to mediated messages. Rather this path assumes that a subset of individuals who are exposed directly to messages engage with others whose behavior is then indirectly influenced. In this scenario, much of the population might not be exposed, or if exposed, might not be directly affected by messages, but may still change behavior because opinion leaders around them are exposed and, in turn, influence their behavior (Katz & Lazarsfeld, 2017). We do not test this path separately in the analyses we present below. However, the choice to use an exogenous measure of coverage means that any observed association between opportunities for exposure and behavior is consistent with this theoretical path. In contrast, if we relied on the association of behavior with individual self-reports of personal exposure to mediated messages, we would have excluded this potential path of effect.

The behavior change literature also offers many hypotheses about how individual exposure to messages may influence behavior; as noted above, three types of beliefs particularly relevant to this study are beliefs about the expected outcomes of behavior, beliefs about descriptive norms, and beliefs about self-efficacy to perform a behavior. While only the first class of beliefs will be directly tested in this study, all three warrant further elaboration. In their reasoned action model, Fishbein and Ajzen assume that mediated messages may persuade individuals about the expected positive (or negative) desirable outcomes for performing a behavior, which influence attitudes towards the behavior and intentions to perform the behavior (2010). For Cialdini et al., mediated messages describe the commonness of a behavior; the more common a behavior is perceived to be, the more likely it is to be adopted (1990). Bandura (among other arguments) claims that valenced messages (and, more so, portrayals of successful enactments of a behavior) increase individuals’ confidence that they can perform a behavior (2001). The primary analyses presented below address, first, the overall association of coverage and intentions. They also present analyses that explore the possibility that the mechanism for the observed effect is through behavioral beliefs, examining the association between media coverage and a summed scale of beliefs about valenced outcomes of smoking.

While the behavioral beliefs pathway of effects is examined here, the test of the pathway through descriptive norm beliefs requires a different dataset. Analysis of the norms path requires coverage estimating the volume of norm information embodied in the coverage; that is, how often smoking is portrayed in the messages (Gibson et al., 2019; Liu et al., 2019) rather than the volume of messages with positive or negative valence. Siegel et al (2022) present evidence for these effects. The third path, through self-efficacy beliefs, would require yet another re-coding of the coverage data focused on, for example, content which addressed how hard or easy it was to quit. That work has not been done.

The approach to design, measurement, and analysis in this study relies on fundamental assumptions about the way media affect behavior. We argued above that important media effects reflect the operation of the full PCE; that when multiple media sources transmit consistent messages, the effects are qualitatively distinct than what might be expected if a single message or a single source was operating. We assume that our summed measures across multiple sources of media content are estimates of the PCE, writ large. The individual source measures which make up those summed measures are conceptually treated as indicators of the PCE. The summed measures then describe what the PCE is saying about tobacco at a given point in time. We do not argue that the messages found on individual sources at a given time are reflective of individual exposure. We do not expect that individual respondents have read a particular tweet or a specific newspaper article included in our content scales. Rather, the summed content scales are treated as indicators of what is available in the communication environment generally through the sources we measured and from the sources we did not include. They reflect potential individual exposure, regardless of which sources an individual actually uses.

We support the reasonableness of this conceptually central assumption by showing that there is substantial homogeneity in trends in tobacco media coverage among measured sources with time as the unit of analysis. The high homogeneity across sources is evidence for the relevance of the individual media sources as indicators of the underlying PCE they are meant to capture. This logic is parallel to evidence of homogeneity across items on any sort of summed scale used to establish construct validity: the scale captures the underlying domain addressed by those items. From this perspective the evidence of homogeneity tests the claim of the existence of a PCE (in this specific domain). More broadly these analyses can suggest PCE measures are worth incorporating in other analyses of media effects.

This PCE conceptual frame for the summed scale has other important implications. We are not hypothesizing effects of individual source content (e.g., of Twitter or of broadcast news) since we do not assume that individuals in our sample are exposed to specific sources. Rather, we assume they are exposed to the whole PCE through direct and indirect media exposure. While other projects might consider individual source effects, we are not testing such hypotheses here.

Further, this framing precludes asking whether those who make more use of any individual source will be more likely to be affected by media content. We cannot argue that people who are heavier users of Twitter, or of broadcast news, are more likely to be exposed to the PCE overall and thus are more likely to be affected by it. It is true that some respondents are more engaged by the PCE overall, and thus more affected by variation over time in its content. However, we do not have any way of assessing that overall engagement in the study. We have self-reports of exposure to some individual sources, but no logical path for combining those to assess tendency to be exposed to the PCE overall. Measures of use of individual sources are not consistently positively associated; we have no logic for weighting them to create an overall scale.

## Prior studies

Notable prior studies have taken a related approach to estimating (non-campaign) media effects on health-related behaviors, measuring media content and health outcomes separately. However, none is a closely matched precedent to this study, which focused on tobacco, used a U.S. national sample of youth and young adults with the day as the unit of analysis, and exploited machine learning methods to capture a census of multiple media sources to describe the PCE.

Tobacco-related studies in this tradition are limited. Pierce and Gilpin (2001) described over time media content trends and behavior trends and pointed to their consistency with cessation attempts but did not test the relationship statistically. Smith et al. (2008) showed a negative relationship between newspaper coverage and youth reports of smoking attitudes and behavior with communities as the unit of analysis. Jamieson and Romer (2015) showed that portrayal of tobacco use in prime time TV dramas predicted increased adult tobacco consumption with time as the unit of analysis. Niederdeppe, Farrelly, Thomas, Wenter, and Weitzenkamp (2007) showed that newspaper coverage of an anti-tobacco campaign predicted reduced youth smoking, with counties as the unit of analysis.

Outside of the tobacco realm, there is a larger evidence base of studies which looked at the ability of media coverage over time to predict health-related outcomes: This should be Fan and Holway (1994) on cocaine use, Stryker (2003) on marijuana use, Dasgupta, Mandl, and Brownstein (2009) on opioid mortality; Yanovitzky and Bennett (1999) and Yanovitzky (2002) on drunk driving, Yanovitzky and Stryker (2001) on binge drinking, Yanovitzky and Blitz (2000) on breast cancer screening, Stevens and Hornik (2014) on HIV testing, Olowokure, Clark, Elliot, Harding, and Fleming (2007), Olowokure et al. (2012) on mumps and influenza diagnostic testing, Hassan (1995) and Schäfer and Quiring (2014) on suicide incidence, Trumbo (2012) on physician visits for influenza, and Niederdeppe and Frosch (2009) on trans-fat product sales. While these studies paralleled the logic of the current study, in that they looked at media coverage association with behavioral outcomes, by and large they limited their estimation of media coverage to that found in one source (the Associated Press wire, or major newspapers) as their indicator of the broader media environment. In contrast, the current study assesses coverage from multiple independent sources.

## The current study

We examine two main effects hypotheses, and one moderation hypothesis. The first hypothesis (H1) is that exposure to tobacco information in the PCE opposed to use (anti) and supportive of use (pro) will affect respondents’ intentions to use combustible cigarettes (with anti-tobacco content reducing intentions and pro-tobacco content increasing intentions). The second main effects hypothesis (H2) is that exposure to such valenced tobacco content will also affect endorsement of beliefs about combustible cigarettes for both smokers and nonsmokers (with anti-tobacco content increasing endorsement of anti-tobacco beliefs and pro-tobacco content reducing endorsement of those beliefs). In addition (RQ1), we ask whether hypothesized effects are moderated by respondent smoking history: will current smokers and current nonsmokers be differentially affected by valenced tobacco content, so that the decision to initiate and the decision not to quit (both of which represent intentions to smoke in the next 6 months) will show different influence of media content? We frame this as a research question because we are uncertain what to expect. Respondents who are current smokers might be paying more attention to tobacco-relevant content given their current behavior, and thus would be more affected by it. Alternately, they may be more committed to their current behavior and thus less vulnerable than nonsmokers to valenced routine media coverage of tobacco that might contain little information novel to them.

## Methods

### Content measures

Between May 2014 and June 2017, the project captured media items from multiple sources about multiple tobacco-related themes on a daily basis. For predicting intentions to smoke combustible cigarettes, we exclude vaping and e-cigarette product items. We were concerned that respondent decisions around combustible cigarette use, which the public health community rejects unequivocally, would be confused by inclusion of media coverage of vaping products with their more ambiguous reception by the public health community. We use a census of tobacco-relevant texts (primarily related to combustible cigarettes) drawn from six media source categories, classified for valence (anti and pro). The media sources include 50 major U.S. newspapers, the Associated Press wire, broadcast news from eight major media outlets, over 100 websites popular either with 12–17- or 18–24-year-olds according to Nielsen, Twitter, and YouTube tobacco-relevant videos. We described the nature of the PCE around tobacco in the days before an individual respondent was interviewed (using both 7- and 28-day measures).

In broad strokes, these were the major steps in this content analysis (Table 1): we chose relevant data archives for each source and then developed keyword search terms to locate broadly relevant tobacco content for each source. We then cleaned each corpus to limit each to truly product-relevant texts using automated coding (i.e., supervised machine learning and dictionary methods), validating our coding with held-aside test sets. We then coded the entire corpus of relevant texts for valence using automated coding.

**Table 1:** Content Sources and Validity Measurements

Source	Period of Measurement	Archive, Method for Locating Eligible Texts	Validity of Product Coding Texts	Method for Assessing Valence	Validity of Coding Valence
Broadcast news	18 May 2014 to 30 June 2017	Lexis–Nexis, 12 keywords	*P* = .97, *R* = 0.98	SML	*P*_pro_ = 0.83; *R*_pro_ = 0.92 *P*_anti_ = 0.86; *R*_anti_ = 0.91
Associated Press	18 May 2014–30 June 2017				
Newspapers	18 May 2014 to 30 June 2017				
Website blogs	18 May 2014 to 30 June 2017	Massachusetts Institute of Technology MediaCloud, 12 keywords			
Twitter	18 May 2014 to 30 June 2017	Gnip Twitter Historical Powertrack, 889 tags, keywords, rules	*P* = .92, *R* = 0.95	SML	*P*_pro_ = 0.92; *R*_pro_ = 0.92 *P*_anti_ = 0.90; R_anti_ = 0.90
YouTube	30 July 2014 to 30 June 2017	YouTube search application program interface, 220 keywords, rules	*P* = .85, *R* = 0.87	SML classification of videos	*P*_pro_ = 0.96; *R*_pro_ = 0.93 *P*_anti_ = 0.60; *R*_anti_ = 0.93

SML, supervised machine learning; P, Precision; R, Recall.

Valence coding took slightly different paths for Twitter and YouTube than for the “long-form” (newspapers, AP, broadcast news, web blogs) texts. In the latter case, we asked sets of nine Amazon Mechanical Turk coders (MTurkers) to classify a sample of 2,400 texts. Coders classified texts as: (a) mostly against the use of tobacco products (anti); (b) mostly supportive of the use of tobacco products (pro); (c) having equal amounts of pro and anti (i.e., mixed); (d) not applicable (i.e., the text is relevant to tobacco but has no valence); or (e) irrelevant to tobacco products. A text was eligible to train the machine learning classifiers if 70% or more of MTurkers (typically nine per text) agreed on its classification. All texts were then re-assigned to two final binary codes: anti or not, and pro or not. Using standard packages in Python, the hand-coded texts were then used to train separate machine classifiers for these two codes, and the developed algorithms were validated on held-out test samples. Validated algorithms were then applied to the entire corpus of relevant texts, with each text assigned both a probability of being pro and a probability of being anti-tobacco. The social media sources (Twitter and YouTube) went through a similar process; however, expert coders rather than MTurkers were used to code the training sample for automated coding (using the same valence definitions in both cases). Also, algorithms were held to similar validation standards during development, although social media items were given binary classifications in the end rather than probabilities.

The analyzed data for the long-form sources were daily sums of the probabilities of all texts from a source being coded anti (or pro). For Twitter, the analyzed data was the simple count of tweets on a given day coded as anti (or pro). For YouTube, the analyzed data were the logged daily count of the number of views in the first 30 days of videos labeled as anti (or pro) being published, with included videos limited to those with more than 10,000 total views in the 180 days after they were first published. Videos were labeled by analyzing all of the available text data associated with the video (i.e., not the visual content). Views were chosen as the daily unit of analysis (rather than number of videos published) because we wanted to weight videos by their likely presence (popularity) in the communication environment. Due to variable lead times for video production and the small number of tobacco-relevant videos published each day, we believe daily views better capture when tobacco was a popular topic in the PCE. Daily views were transformed into (natural) logged versions because there were extreme spikes in numbers of views on days when popular videos were highly viewed.

In addition to coding long-form texts for themes and valence, we differentiated texts that only dealt with the topic in passing (e.g., mentioning that someone was holding a cigarette, but not otherwise mentioning smoking) from those where tobacco was a more substantial issue for the text (Gibson et al., 2019). Assessment of valence could only be applied to “more than passing mention” long-form texts and only they are included in the analyses reported below. In contrast, all tweets coded as relevant to the topic were also coded for valence and treated as “more than passing mention” texts. As there was a 140-character limit on tweets for most of the study period, any mention of tobacco in a tweet would constitute a substantial portion of its content. Similarly, the YouTube relevancy classifier was also built to find “more than passing mention” videos, so all videos were so coded.

Precision (what proportion of the coded sample was “true”) and recall (what proportion of the “true” texts in the corpus were located through the search process) estimates were calculated for each source category to establish that the coding was valid, comparing machine coding with hand coding serving as the gold standard. Table 1 presents all of the precision and recall statistics. While there was some variation, in nearly every case, precision and recall were above 0.80. The only exception was precision of YouTube anti-tobacco coding (0.60). This content analysis process is fully detailed elsewhere (Gibson et al., 2019; Kim et al., 2020).

### Survey measures

The survey sample was designed to represent the U.S. population of youth and young adults between 13 and 25 years of age. Participants were first surveyed by landline or cell phone from June 2014 to June 2017 (*N* = 11,847, American Association for Public Opinion Research #3 Response Rate = 21%). Thirty-eight percent of eligible participants completed a second interview six months after their first one (*n* = 4,470). The sampling and weighting procedures were developed, and the survey was implemented by Social Science Research Solutions.

The survey-derived measures used in the analyses below include a measure of current smoking status, intentions to initiate and (not) quit smoking in the next 6 months, and a scale averaging beliefs about the consequences of smoking combustible cigarettes.

#### Established smoking

Respondents were asked “Have you ever tried smoking cigarettes, even one or two puffs?”, “Have you smoked at least 100 cigarettes, which is 5 packs, in your entire life?”, “During the past 30 days, on how many days did you smoke cigarettes?” and categorized as established current smokers if they answered “yes” to the first two questions and more than zero to the third question.

#### Intentions

Established smokers were asked: “How likely is it that you will try to quit smoking completely in the next 6 months? By completely, I mean not smoking tobacco cigarettes at all. Would you say definitely will not, probably will not, probably will, or definitely will?” The rest of the respondents were asked: “How likely is it that you will smoke a tobacco cigarette, even one or two puffs, at any time in the next 6 months? Would you say definitely will not, probably will not, probably will, or definitely will?” The measure in the analyses reported below combines these measures: nonsmokers who said anything other than “definitely not” about initiation, and smokers who said anything other than definitely yes about quitting intention, were coded as one; the other respondents were coded as zero. Thus, the measure is 1 when a respondent has some intention of smoking in the next 6 months and 0 otherwise.

The first hypothesis uses intentions rather than behavior as the outcome. While a behavioral outcome might be preferred as a “harder” measure, the logic of our analyses precluded its use. We used content available to an individual in the 7 or 28 days before the day of interview as our predictors. The behavioral measures asked about tobacco use over the prior 30 days. The intention measures asked about future behavior. If we had used the behavioral measures as the outcome, the temporal order between the content and the behavior would have been confused. We could not have been sure that the exposure happened before the behavior. Given that the intentions measures asked about the future, the temporal order between pre-survey content and intentions for post-survey expected behavior was more straightforward.

These intention measures are substantially related to behavioral outcomes. As noted above, for 38% of the baseline respondents, we have follow-up questionnaires administered about 6 months after the first interview. They were asked about their smoking in the previous 30 days. For the 3,940 nonsmokers at baseline who were recontacted, 79% had said they definitely did not intend to initiate smoking in the next 6 months and only 2.1% of them self-reported 30-day smoking 6 months later. In contrast, for those who expressed a positive intention (probably will or definitely will), 48% reported smoking (significantly more, *p* < .001). For the 441 smokers retained in the follow-up study, there were 233 who said they would probably or definitely quit; 87.5% of them reported a quit attempt in the 6 months prior to the recontact survey. Among those who had said they would definitely not quit, only 25% reported trying to quit in the 6 months prior to recontact. This difference was also statistically significant (*p* < .001).

#### Beliefs

All respondents were asked “The next set of questions is about tobacco cigarettes. I’ll read a statement, then please tell me whether you strongly disagree, disagree, agree, or strongly agree with it. If I smoke every day, I will … develop headaches.” Other beliefs included: … develop sexual and/or fertility problems, … develop cancer, … get wrinkles, … lose my teeth, … get yellow fingers, … become addicted to nicotine, … be controlled by smoking, … look uncool, [It will] be a turn off to other people, … feel relaxed (reversed), … enjoy life more (reversed). Two items were reverse-coded so that all items had the most anti-smoking belief on the same end of the scale. The beliefs were asked in random order. An averaged anti-smoking belief scale was highly internally consistent; Cronbach’s alpha is 0.87.

### Analyses

Each respondent was assigned anti- and pro-tobacco media exposure scores for each of the six sources, summing coverage over the previous seven days in primary analyses. We examined an alternative specification of previous 28 days in sensitivity analyses. For both conceptual and statistical reasons, we aggregated across the set of six media sources for anti- and pro-content using principal component analysis and assessed their internal consistency. As noted above, we were interested in two aspects of the PCE: the quantity of coverage and the consistency among sources. Statistically, we focused on their common core because each individual source measure can be interpreted as a measure with some error of the PCE. Aggregation creates a new measure with reduced error. Also, examining a reduced number of coverage measures allows the analysis to focus on fewer parameters, and thus limits the likelihood of finding significant results by chance. After weighting each source by principal component weights, the aggregated measure reflects the consistency of the sources. A priori, we decided to follow a common path for the anti- and pro- sources to maximize comparability.

About 10 respondents were interviewed on each of the 1,140 days; all those interviewed on a given day were assigned the same media exposure score. The hypotheses were tested with logistic regression, predicting intention from the media exposure measures, along with measures for smoking status, and time. We included smoking status because it was likely to be a strong predictor of intention to smoke and thus its inclusion might allow us to see the effects of media exposure more clearly.

Other survey measures should not be potential confounders, since each week’s sample was randomly selected from the population so the exogenous measure of coverage should be independent of respondent characteristics. Thus, while those other characteristics (e.g., age, race-ethnicity, gender) might be associated with the outcomes (intention and beliefs), they logically cannot account for the association of exogenously measured content and those outcomes. We did adjust for time (days after the start of the study) recognizing that there might be secular trends for both intentions and media coverage independent of their influence on each other.

The sample is weighted both to adjust for the sampling design and to match the U.S. Census Bureau’s American Community Survey on major demographic variables to improve representativeness. Analyses are clustered by interview date given that the content is identical within each daily cluster. The use of weights and clustering required generating appropriate adjusted standard errors. The results section presents univariate descriptive information for media content and survey variables; results of the principal components factor analyses for the media content variables; examination of main effects of coverage on intentions; exploration of whether smoking status moderates the effects of coverage on intentions; main effects of coverage on beliefs; exploration of whether smoking status moderates the effects of coverage on beliefs; and summarizes three sensitivity analyses: prior 28-day coverage, multilevel models, and models excluding YouTube views. All analyses were undertaken with Stata (version 14).

## Results

Table 2 presents the univariate information for all of the included variables. The sources vary widely in how many texts they contribute to the analyses. The anti-tobacco texts dominate the first four sources, anti- and pro-tobacco videos are about equal for YouTube views, and anti-tobacco tweets are about half as common as pro-tobacco tweets for Twitter.

**Table 2:** Univariate Information for All Included Variables

Variable	Mean (SD)	Mean (SD)
Source—past seven days	Anti-tobacco texts	Pro-tobacco texts
Broadcast news	0.20 (0.22)	0.05 (0.10)
Associated Press (AP)	0.93(0.44)	0.05 (0.07)
Newspapers	3.62 (1.28)	0.57 (0.33)
Websites	5.31(1.94)	1.07 (0.56)
Twitter	12,948 (3,457)	21,336 (5,546)
YouTube views	79,773 (96,229)	72,317 (54,640)
Intention to smoke among nonsmokers (% some intention)	22% (*n* = 10,369)	
Intention not to quit among smokers (% some intention not to quit)	80% (*n* = 1,422)	
Intention open to smoking among all	29% (*n* = 11,791)	
Established smokers (lifetime more than 100 and current)	12% (*n* = 11,847)	
Anti-tobacco belief scale (1–4) mean (*SD*)	3.00 (0.43) (*n* = 11,847)	

*Note:* Sources were summed for 7 days prior to interview date. The reported numbers represent the total pro- or anti-tobacco coverage in each source over that period. The means are estimated at the person-level. Survey measures reflect population weighting.

Overall, the four long-form sources and Twitter formed a coherent primary factor, although Twitter was more associated with the anti-tobacco coverage primary factor than with the pro-tobacco coverage factor. The YouTube views measures were minimally associated with the anti and pro factors. Specifically, the five anti-source variables were weighted together with the following factor weights: Associated Press (0.44), Broadcast (0.39), newspapers (0.49), web (0.50), and Twitter (0.41), while anti-YouTube views were negatively associated with the common factor. The theta (a parallel measure to Cronbach’s alpha that captures the internal consistency for items summed according to their component weights from a principal component factor analysis; Armor, 1974) for the combined five-item index is 0.77. For the five pro-source variables the primary factor weights are Associated Press (0.40), Broadcast (0.49), newspapers (0.54), web (0.45), and Twitter (0.33), and the theta is 0.79. Pro-YouTube views are again negatively associated with the other sources. These analyses led us to create two anti and two pro variables. The first in each set incorporates all of the long-form sources plus Twitter; it is a standardized measure (mean = 0, standard deviation = 1). The second variable in each set is the standardized YouTube measure alone, since it did not positively covary with the other source measures.

### Test of hypotheses about the intentions outcome (H1 and RQ1)

**Figure 1:**
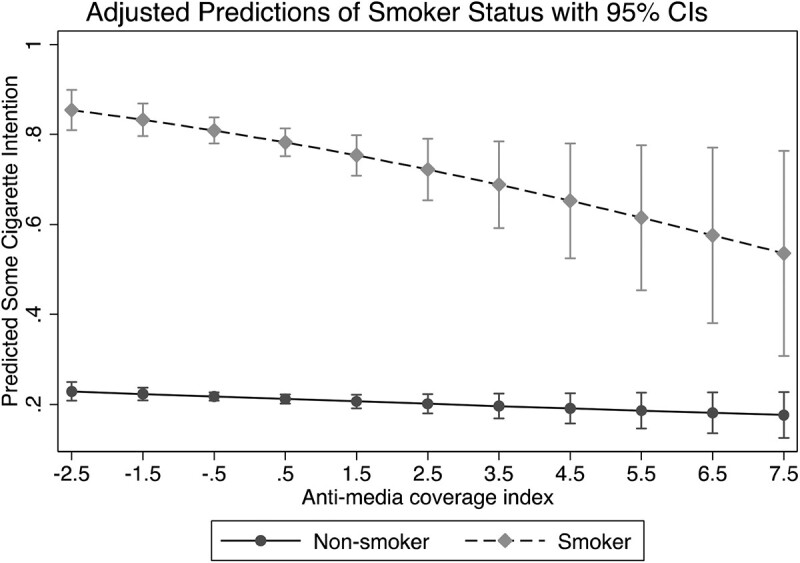
Adjusted predictions of smoker status with 95% CIs.

Table 3 presents the main effects and significant moderation analyses. The anti-tobacco media coverage index showed a significant negative association (Odds ratio (OR) = 0.95, *p* = .03, 95% confidence interval (CI) = 0.91–1.00) with intentions to smoke, such that, as expected for H1, more anti-tobacco media coverage is associated with lower intentions to smoke. The other media variables were not significantly related to intentions to smoke, although their signs (negative for the views of anti-tobacco YouTube coverage, positive for the two pro-tobacco media content measures) were consistent with the expected directions. Regarding RQ1, there was one significant moderation effect: the anti-tobacco media coverage variable interacted negatively with smoker status (OR = 0.88, *p* = .04, 95% CI = 0.77–1.00), suggesting that the expected negative effects of anti-tobacco coverage on intentions to smoke were larger for smokers than for nonsmokers. Figure 1 illustrates the predicted effects separately for established smokers and nonsmokers.

**Table 3:** Effects of Media Coverage on Intentions to Smoke, Main Effects, and Interaction

Predictor	Odds Ratios: Main Effects Model	Confidence Interval (CI)	Odds ratios: Model with Smoking Status Interaction	CI
Standardized time	0.92	0.83–1.02	0.92	0.83–1.02
**Established smoking status**	**14.21**	**11.84**–**17.06**	**14.26**	**11.87**–**17.12**
**Anti-media coverage index**	**0.95**	**0.91**–**1.00**	0.97	0.93–1.01
Pro-media coverage index	1.02	0.99–1.05	1.02	0.99–1.05
Anti-YouTube views	0.96	0.89–1.04	0.96	0.89–1.04
Pro-YouTube views	1.04	0.95–1.12	1.04	0.95–1.13
Established smoking status* Anti-media coverage index	—	—	**0.88**	**0.77**–**1.00**
Constant	0.28	0.26–0.29	0.28	0.26–0.29
*N*	11,343		11,343	

*Note:* Logistic regression, clustered by date, significant (*p* < .05) predictors bolded. The media coverage index is a standardized scale made up of Associated Press, Broadcast news, newspapers, websites, and Twitter. YouTube views are logged. Smoking status was only a significant moderator for the anti-media coverage index.

*It indicated that this is an interaction term -- the product of Established smoking status and the Anti-media coverage index.

### Tests of hypotheses about the beliefs outcome (H2)

H2 proposed that the media coverage variables would influence beliefs about tobacco—that periods of more anti-tobacco coverage or views would be associated with increased anti-tobacco beliefs while periods of more pro-tobacco coverage or views would have the opposite effect. The results of the ordinary least squares regression analysis are presented in Table 4. The set of media coefficients accounted for no significant variance in the belief scale and none of the individual media coefficients were significant at the *p* < .05 level (views of anti-tobacco YouTube content was close at *p* = .051). Also, there was no evidence for interactions of the seven-day media coverage variables with smoker status.

**Table 4:** Effects of Media Coverage on Anti-Smoking Beliefs, Main Effects

Predictor	Model with Main effects	
	B	Confidence interval
Standardized time	0.017	**−**0.004 to 0.038
Established smoker	**−0.409**	**−0.444 to −0.373**
Anti-media coverage index	**−**0.004	**−**0.012 to 0.005
Pro-media coverage index	0.002	−0.005 to 0.008
Anti-YouTube views	−0.014	−0.028 to 0.000
Pro-YouTube views	−0.002	−0.020 to 0.016
Constant	3.106	3.096 to 3.117
*N*	11,381	

*Note:* Ordinary least squares regression, clustered by date, significant (*p* < .05) predictors bolded. The media coverage index is a standardized scale made up of Associated Press, Broadcast news, Newspapers, Websites, and Twitter. YouTube views are logged. Smoking status was not a significant moderator of any media coverage variables.

Sensitivity analyses using: (a) prior 28-day coverage; (b) an alternative approach to clustering, multi-level models clustered on date; and (c) excluding YouTube coverage showed substantively similar results. We chose the seven-day analysis as our primary focus, consistent with prior studies which suggested that routine content effects have a half-life of a day (Fan, 2002). However other research, particularly examining effects of advertising content, permits longer lagged effects (Southwell, 2002). Similar to the seven-day findings, for the 28-day media coverage, theta is 0.85 for the anti-sources and 0.79 for the pro-sources. YouTube views are negatively associated with the common factors. Among the media variables, only the anti-media index is a significant predictor of intentions (OR = 0.94, *p*= .02, 95% CI = 0.90–0.99);(see Supporting Tables S1 and S2). For the moderation models, the interaction of the 28-day anti-tobacco media index with smoker status is also significant (OR = 0.86, *p* = .01, 95% CI = 0.77–0.97). There was significant evidence for an unexpected effect of the 28-day measure of views of anti-tobacco YouTube coverage. While none of the other 28-day media coverage variables were significantly associated with the belief scale, the coefficient for the YouTube anti-tobacco variable was (*b* = −0.03, *p* = .002, 95% CI = − 0.04, −0.01), consistent with the tendency for 7-day views of anti-tobacco YouTube coverage to predict anti-tobacco beliefs. Multi-level models clustering on date showed very similar results, though the interaction with smoking status did not reach significance, the point estimate was very similar (see Supporting Tables S3 and S4).

Finally, the available sample is reduced because the YouTube views data are not available for around 450 cases (YouTube data was only collected starting 6 weeks after the other media data). If the YouTube variables are not included in the model, the power increases, but the coefficient remains the same: Main effects 7-day model: OR = 0.95, *p* = .01, 95% CI = 0.92–0.99) (see Supporting Tables S5 and S6).

## Discussion

Anti-tobacco 7-day media coverage is significantly associated with survey-measured lower intentions to smoke among the U.S. youth and young adult population. The effect was the same in sensitivity analyses using the 28-day version of the media coverage variable, excluding the nonsignificant YouTube media variables, and using a multilevel model. This effect was stronger among current smokers than among nonsmokers. There were no parallel effects for either measure of pro-tobacco coverage or for views of anti-tobacco videos on YouTube. Also, we saw no consistent association between media and smoking-related beliefs (although there were hints of an anomalous effect of views of anti-tobacco YouTube videos decreasing anti-tobacco beliefs).

To the best of our knowledge, this is the largest test of a media effects hypothesis showing the relationship between an exogenous measure of media coverage and a survey measure of a behavior-related outcome. It used daily media coverage data for six new and old media sources over 3 years, and a matched in time rolling cross-sectional survey of 11,847 youth and young adults. This study is only possible because of the advent and growth of electronic media archives, and the development of methodological tools that support machine coding of massive text corpuses. While there are many studies that report content analyses (including about tobacco) and others which report survey results, the opportunity to merge the two data streams on this scale has been of particular value in moving toward causal claims. The research design incorporated the use of independent measurement methods for collecting the media and the outcome data, the use of time as the unit of analysis, and the use of prior coverage to predict current intentions. All of these features increase confidence that the observed association shows the influence of coverage on intentions.

The value of the data analyzed here is their generalizability. At the outset of the article, we stated our intention to capture effects of the messy communication environment and to do so in an ecologically realistic way. The media coverage measure is a census of the six included sources for the 3-year time period, and we argue that this coverage is an indicator of the full PCE. The substantial inter-source consistency in variation over time of valenced tobacco messaging across five of the six sources (as shown by the principal component analyses) is evidence for the presence of common message themes across those media sources. Similarly, the survey is designed to be nationally representative of U.S. youth and young adults, the population most vulnerable to smoking onset. In focusing on the association between coverage and survey responses, we allow a range of paths of effect to operate; these include belief change resulting from direct exposure by respondents but also social diffusion of content from media to those who are directly exposed and on to those with whom they share content. While we saw successful prediction of intentions, we were not able to attribute it to mediation by belief change since we were not able to show that the PCE predicted beliefs.

The analysis detected an effect in the expected direction for the intentions outcome for the composite anti-tobacco index. However, there was thinner evidence for the pro-tobacco index, or for the pro- and anti-YouTube measures. We know that there was relatively little pro-tobacco content in sources other than Twitter and YouTube (Table 2) so the failure to find an influence of pro-tobacco content from the composite index is perhaps unsurprising. YouTube video view effects were examined separately given the lack of association with the other sources, suggesting it was not a strong indicator of the PCE. If YouTube was to have effects, it would likely reflect exposure to that channel. However, although the great majority (86%) of survey respondents reported weekly use of YouTube, we speculate that it is unlikely that very many saw the specific anti- and pro-tobacco videos measured in a given week. There were about 80,000 anti-tobacco YouTube views in an average week in the entire population. There are about 55,000,000 youth between the ages of 13–25 years. Even under the extreme assumption that all views were from 13- to 25-year-olds, the average respondent in our sample would only be expected to view 0.01 anti-tobacco videos weekly. This probability of viewing is too low to expect any effects of YouTube in isolation. The mostly null evidence for coverage effects on the belief index may reflect already well-entrenched views of smoking in this population. On the 1–4 anti-smoking belief scale, the mean of 3.0 varied little week to week (weekly standard deviation = 0.07).

## Limitations

In general, the coefficients we report are absolutely small, even though the sample sizes are large. Still the models tested are few, the hypotheses stated a priori, and the effects are significant and not sensitive to alternative specifications. This reduces concerns about the possibility that the results happened by chance.

In addition, the small size of the observed effects should perhaps be seen as unsurprising. The content analyses of six old and new media source categories are presented as indicators of what is present in the PCE. They are not comprehensive, however, and other indicators may tell a different story. Also, the assessment of text valence reflects an imperfect supervised machine learning process. Although the recall and precision estimates from this coding were good, though less than ideal, random error in our supervised machine learning process may have added noise to weekly estimates of valenced coverage. Also, the content measures represent opportunities for exposure writ large, but do not assess actual respondent exposure for the youth and young adults in the sample. There were relatively few substantial tobacco-related stories documenting new information about tobacco risks over the 3-year period so, while there was week-to-week variation in coverage, the effects observed here may underestimate the effects detectable if there was more variation in content. Crucially, tobacco content represents a very small proportion of the content of these sources (and of the PCE generally). Responsiveness to the observed variation in tobacco-relevant media coverage may well be dwarfed given the context of so much other content. Similarly, the outcome variables, binary intention to smoke (weekly mean = 0.29, standard deviation = 0.06) and particularly smoking beliefs, as noted above, did not vary greatly over time; information about the risks of smoking is widely diffused and both intentions and beliefs are likely to be stable. We suspect that all of these limitations made it harder to detect the observed hypothesized effects. It is also important to emphasize that this is a population level estimate; a small effect found for the entire population can signal a large aggregate change.

As noted above, some might expect analyses which focus on individuals who are personally more exposed to the named media sources to show bigger effects of the coverage variables. We do have survey measures of individual use of specific sources. However, our conceptual logic treats specific source coverage estimates only as indicators of the PCE. We do not have a measure of exposure to the full PCE, and no way to combine the individual source use measures to create such an overarching measure that would be required for such an analysis.

In this study, we focus on the effects of the PCE on smokers’ and nonsmokers’ combustible cigarette intentions. A causal claim about such an association relies on the credibility of two assumptions. First, we assume that exogenously measured media content is not caused by individual intentions. We assume that week-to-week variation in media attention to tobacco reflects external events and is not a direct response to population changes in intentions to use cigarettes. Second, we assume that other variables do not have independent effects on both media source coverage and individual intentions. For example, while we would expect that official announcements or releases of damaging research studies would affect media coverage and respondent intentions, we assume media coverage would represent the intervening process in such effects—people would not know about such announcements or studies without media coverage of them. We think these are reasonable assumptions in the specific context of the current study. Still, the credibility of inferences from analyses based on observed associations of the exogenously measured PCE and survey-measured outcomes depends on the credibility of these assumptions in a specific context.

## Conclusions

In this novel study, we show evidence that variation in valenced media coverage of tobacco predicts youth and young adult intentions to smoke (and we know also that such intentions strongly predict smoking behavior over the subsequent 6 months, Jesch, Kikut, & Hornik, 2021). This study has methodological importance, given how difficult it is to mount studies that can make credible claims for media effects in ecologically representative contexts. The study has substantive importance as well. There is a strong literature showing that deliberate anti-tobacco campaigns can affect smoking behavior (Farrelly et al., 2017; Farrelly, Nonnemaker, Davis, & Hussin, 2009; Huang et al., 2017; Niederdeppe, Farrelly, & Haviland, 2004), but evidence that variation in routine media coverage also affects behavior-relevant outcomes is more rare. While anti-tobacco campaigns have had a substantial presence in the PCE at times, we suspect that for most people, day-to-day direct and indirect exposure to routine coverage of tobacco in media is more frequent over the long run. Then it is likely that routine media coverage of tobacco may matter as much or more in affecting tobacco related decisions. Still, it was possible that routine coverage of tobacco, not designed to be persuasive, could have been overwhelmed by the massive amount of other content flowing through the media environment. But our results provide evidence that routine coverage does matter; variation in (anti-)valenced tobacco coverage in the PCE does predict (non-) intentions to engage in tobacco use.

This study is specifically about media effects on tobacco, but as elaborated in the sections that point to underlying substantive considerations, we would argue that the study has substantial relevance to communication theory and methods more generally. In these final paragraphs, we offer a case for that relevance claim. The central idea of this project is its call for examining the effects of the broad PCE. Media research, regardless of the domain, will benefit from recognizing that people are immersed in both intentionally persuasive and simply descriptive messages from multiple sources. Media effects research should recognize that people live in such complex communication environments; they process exposures to messages from mediated and personal sources over time and those messages and sources provide sometimes consistent and sometimes inconsistent content. Individuals bring varying mental frameworks and social circumstances to those exposures. Also, the mechanisms of effect may include individual exposures, but can also reflect institutional and social paths of effect which do not assume individual exposure. One task for media research then is to study the effects that happen in such complex contexts, recognizing that this may come at the cost of being unable to isolate individual message and source effects, even though isolation of effects can be sometimes appropriate. However, the study of such PCE effects can make little progress without appropriate methods.

The commitment to the study of media effects in ecologically representative contexts makes substantial demands regarding appropriate methods. A study needs to measure the PCE and an outcome of interest with a unit of analysis permitting observation of the association between the two. Simultaneously it must minimize the threat of other explanations for the association which might undermine causal claims.

As noted above, there are multiple ways that people have addressed this issue. We argue that the approach taken here has some benefits over prior approaches, despite other drawbacks, and is applicable across substantive areas. Specifically, we capture the PCE with an exogenous measure of opportunities for exposure, limiting prior concerns about bias due to a common method for independent and dependent variables. We use time as the unit of analysis, comparing across periods when the nature and volume of content varies. We survey rolling cross-sectional samples to equally represent the population at each unit of time; because the samples at each time period are different only by chance, we can be confident that individual characteristics cannot explain any observed association between the PCE and an outcome measure.

A particularly hard problem is establishing that a measure of the PCE is adequate. It is not feasible to census all available messages that an individual might be exposed to, partly because many such messages and sources are difficult or impossible to measure. Underpinning the work presented here is a fundamental measurement hypothesis. We assume that if we collect a census of relevant content from a range of sources that are accessible through public databases that these sources can serve as useful indicators of the underlying PCE, including the elements of the PCE that are not accessible in such databases. This idea is only partially testable; we show that most of the sources we capture are consistent over time, when one source reports more content of a specific sort (in this case, anti-tobacco content) the others do as well. This observed coherence among measured sources provides evidence that they are measuring the same underlying PCE (and that their sum will provide a substantially reliable assessment of the PCE). We cannot test the further assumption that the PCE they capture also reflects the full PCE including unmeasured sources, so that remains an assumption.

We also have summarized our approach to a second difficult problem in describing the PCE—coding a census of content from multiple sources which can include millions of individual messages. Our approach combined hand coding of sample items by multiple coders with machine learning algorithms and formal validity testing. This approach may be useful for other research.

In some ways, our effort here is still an early effort both substantively and methodologically to solve what we think is a central problem in media research: understanding the effects of the PCE on particular outcomes. Our observed effects are small, perhaps unsurprisingly given the tenuous relationship between opportunities for exposure in a narrow domain and likely actual exposures, and the expected impact of such exposures relative to direct experience. Development of these ideas and methods is made more urgent by the proliferation of mediated sources and individual differences in exposure patterns. There is much more work to be done to apply these ideas in other domains, as well as to develop these methods and others relevant to assessing PCE effects.

## Funding

Research reported in this publication was supported by the National Cancer Institute of the National Institutes of Health (NIH) and Food and Drug Administration (FDA) Center for Tobacco Products under Award Number P50CA179546. The content is solely the responsibility of the authors and does not necessarily represent the official views of the NIH or the FDA.

*Conflict of Interest*: none declared.

## Supporting information

Supporting information are available in the online version of this article.

## Supplementary Material

jqab052_Supplementary_Tables
