# Supplementary material for: The Effects of Tobacco Coverage in the Public Communication Environment on Young People’s Decisions to Smoke Combustible Cigarettes
Source: J Commun. 2022 Jan 13;72(2):187–213. doi: 10.1093/joc/jqab052 (PMC8974361; doi:10.1093/joc/jqab052)
Supplement: jqab052_Supplementary_Tables [file jqab052_supplementary_tables.docx]

Supplemental tables:

Supporting Table S1

*Effects of media coverage on intentions to smoke, main effects and interaction (28 day version)*

| Predictor | Odds Ratios: Main effects model | CI | Odds ratios: Model with smoking status interaction | CI |
| --- | --- | --- | --- | --- |
| Standardized time | 0.89 | 0.78, 1.01 | 0.89 | 0.78, 1.01 |
| **Established smoking status** | **14.21** | **11.83, 17.05** | **14.30** | **11.91, 17.18** |
| **Anti-media coverage index** | **0.94** | **0.90, 0.99** | 0.96 | 0.92, 1.01 |
| Pro-media coverage index | 1.00 | 0.97, 1.04 | 1.00 | 0.97, 1.04 |
| Anti-YouTube views | 1.00 | 0.92, 1.09 | 1.00 | 0.91, 1.08 |
| Pro-YouTube views | 0.99 | 0.90, 1.10 | 0.99 | 0.90, 1.10 |
| Established smoking status* Anti-media coverage index | -- | -- | **0.86** | **0.77, 0.97** |
| Constant | 0.27 | 0.26, 0.29 | 0.28 | 0.26, 0.29 |
| N | 11,343 |  | 11,343 |  |

*Note:* logistic regression, clustered by date, **significant (*p*<.05) predictors bolded**. The media coverage index is a standardized scale made up of AP, Broadcast news, Newspapers, Websites, and Twitter for the past 28-days (instead of 7-days). YouTube views are logged. Smoking status was only a significant moderator for the Anti-media coverage index.

Supporting Table S2

*Effects of media coverage on anti-smoking beliefs, main effects (28 day version)*

| Predictor | Model with Main effects | |
| --- | --- | --- |
|  | B | CI |
| **Standardized time** | **0.039** | **0.011, 0.066** |
| **Established smoker** | **-0.408** | **-0.444, -0.373** |
| Anti-media coverage index | 0.008 | -0.002, 0.017 |
| Pro-media coverage index | 0.005 | -0.002, 0.012 |
| **Anti-YouTube views** | **-0.026** | **-0.043, -0.010** |
| Pro-YouTube views | 0.003 | -0.015, 0.022 |
| Constant | 3.107 | 3.096, 3.117 |
| N | 11,381 |  |

*Note:* OLS regression, clustered by date, **significant (*p*<.05) predictors bolded**. The media coverage index is a standardized scale made up of AP, Broadcast news, Newspapers, Websites, and Twitter for the past 28-days (instead of 7-days). YouTube views are logged. Smoking status was not a significant moderator of any media coverage variables.

Supporting Table S3 –.

*Effects of media coverage on intentions to smoke, main effects and interaction* Multi-level model clustered on date

| Predictor | Odds Ratios: Main effects model | CI | Odds ratios: Model with smoking status interaction | CI |
| --- | --- | --- | --- | --- |
| Standardized time | 0.92 | 0.82, 1.02 | 0.92 | 0.83, 1.02 |
| **Established smoking status** | **15.18** | **12.54, 18.37** | **15.21** | **12.56, 18.42** |
| **Anti-media coverage index** | **0.95** | **0.91, 1.00** | 0.97 | 0.92, 1.01 |
| Pro-media coverage index | 1.02 | 0.99, 1.05 | 1.02 | 0.99, 1.05 |
| Anti-YouTube views | 0.97 | 0.90, 1.05 | 0.97 | 0.90, 1.05 |
| Pro-YouTube views | 1.03 | 0.94, 1.13 | 1.03 | 0.94, 1.13 |
| Established smoking status* Anti-media coverage index | -- | -- | 0.88 | 0.77, 1.00 |
| Constant | 0.27 | 0.25, 0.28 | 0.27 | 0.25, 0.28 |
| Estimated variance for date cluster | 0.13 | 0.08, 0.21 | 0.13 | 0.08, 0.21 |
| N | 11,343 |  | 11,343 |  |

*Note:* Mixed-effects logistic regression, clustered by date, **significant (*p*<.05) predictors bolded**. The media coverage index is a standardized scale made up of AP, Broadcast news, Newspapers, Websites, and Twitter for the past 7-days. YouTube views are logged. Smoking status was a marginally significant moderator for the Anti-media coverage index (*p*=0.051).

Supporting Table S4 – multi-level model clustered on date

*Effects of media coverage on anti-smoking beliefs, main effects*

| Predictor | Model with Main effects | |
| --- | --- | --- |
|  | B | CI |
| Standardized time | 0.018 | -0.003, 0.039 |
| **Established smoker** | **-0.407** | **-0.441, -0.373** |
| Anti-media coverage index | -0.003 | -0.011, 0.006 |
| Pro-media coverage index | 0.003 | -0.003, 0.009 |
| **Anti-YouTube views** | **-0.015** | **-0.029, 0.000** |
| Pro-YouTube views | -0.003 | -0.020, 0.015 |
| Constant | 3.105 | 3.094, 3.116 |
| Estimated residual variance for date cluster | 0.189 | 0.182, 0.196 |
| N | 11,381 |  |

*Note:* OLS regression, clustered by date, **significant (*p*<.05) predictors bolded**. The media coverage index is a standardized scale made up of AP, Broadcast news, Newspapers, Websites, and Twitter for the past 28-days (instead of 7-days). YouTube views are logged. Smoking status was not a significant moderator of any media coverage variables.

Table S5.

*Effects of media coverage on intentions to smoke, main effects and interaction*

| Predictor | Odds Ratios: Main effects model | CI | Odds ratios: Model with smoking status interaction | CI |
| --- | --- | --- | --- | --- |
| **Standardized time** | **0.92** | **0.86, 0.98** | **0.92** | **0.86, 0.98** |
| **Established smoking status** | **14.34** | **11.99, 17.15** | **14.56** | **12.15, 17.44** |
| **Anti-media coverage index** | **0.95** | **0.92, 0.99** | 0.96 | 0.93, 1.00 |
| Pro-media coverage index | 1.02 | 0.99, 1.06 | 1.02 | 0.99, 1.06 |
| Established smoking status* Anti-media coverage index | -- | -- | 0.91 | 0.82, 1.01 |
| Constant | 0.28 | 0.26, 0.29 | 0.28 | 0.26, 0.29 |
| N | 11,793 |  | 11,793 |  |

*Note:* logistic regression, clustered by date, **significant (*p*<.05) predictors bolded**. The media coverage index is a standardized scale made up of AP, Broadcast news, Newspapers, Websites, and Twitter. Smoking status was not a significant moderator for any of the media coverage variables. It is included here for direct comparison with the main analyses.

Table S6.

*Effects of media coverage on anti-smoking beliefs, main effects*

| Predictor | Model with Main effects | |
| --- | --- | --- |
|  | B | CI |
| Standardized time | 0.010 | -0.012, 0.014 |
| **Established smoker** | **-0.411** | **-0.445, -0.377** |
| Anti-media coverage index | -0.003 | -0.011, 0.005 |
| Pro-media coverage index | 0.002 | -0.005, 0.008 |
| Constant | 3.110 | 3.100, 3.121 |
| N | 11,831 |  |

*Note:* OLS regression, clustered by date, **significant (*p*<.05) predictors bolded**. The media coverage index is a standardized scale made up of AP, Broadcast news, Newspapers, Websites, and Twitter. Smoking status was not a significant moderator of any media coverage variables.
